# Prevalence of cognitive impairment and its associated factors in type 2 diabetes mellitus patients with hypertension in Hunan, China: a cross-sectional study

**DOI:** 10.3389/fpsyt.2024.1445323

**Published:** 2024-12-19

**Authors:** Hongying Liu, Ziling Feng, Wenyan Zhang, Yamin Liu, Ni Xiong, Wenhang Chen, Jianzhou Yang, Xin Yin Wu, Zeya Shi, Wenjie Dai

**Affiliations:** ^1^ Case Room, Medical Department, Hunan Prevention and Treatment Institute for Occupational Diseases, Affiliated Prevention and Treatment Institute for Occupational Diseases of University of South China, Changsha, Hunan, China; ^2^ Department of Epidemiology and Health Statistics, Xiangya School of Public Health, Central South University, Changsha, Hunan, China; ^3^ Department of Nephrology, Xiangya Hospital, Central South University, Changsha, Hunan, China; ^4^ Department of Preventive Medicine, Changzhi Medical College, Changzhi, Shanxi, China; ^5^ Nursing Department, Hunan Prevention and Treatment Institute for Occupational Diseases, Affiliated Prevention and Treatment Institute for Occupational Diseases of University of South China, Changsha, Hunan, China

**Keywords:** type 2 diabetes mellitus, hypertension, cognitive impairment, prevalence, associated factors

## Abstract

**Background:**

Individuals with both type 2 diabetes mellitus (T2DM) and hypertension have an increased risk of cognitive impairment (CI) compared to those with either T2DM or hypertension. Therefore, this study aims to identify the prevalence of CI and its associated factors in T2DM patients with hypertension in Hunan, China.

**Methods:**

This cross-sectional study included T2DM patients with hypertension admitted to the Department of Endocrinology of Yuanjiang People’s Hospital in Hunan Province from July 2022 to February 2023. Data on sociodemographic, lifestyle, disease-related characteristics, and cognitive function were collected through face-to-face interviews. Cognitive function was assessed using the Mini-Mental State Examination. Backward stepwise multivariable logistic regression analysis was used to identify factors associated with CI. The predictive power was examined using the receiver operating characteristic (ROC) curve.

**Results:**

A total of 475 participants were included. The prevalence of CI was 38.9% (95% confidence interval [95% CI]: 34.5%–43.3%). Multivariable logistic regression analysis showed that advanced age (odds ratio [OR]=3.21, 95% CI: 1.74–5.93), elementary school or below (OR=3.51, 95% CI: 1.19–10.31), per capita monthly household income ≤2000 RMB (OR=5.29, 95% CI: 2.66–10.51), not current reading books or newspapers (OR=4.48, 95% CI: 1.26–15.99), not current playing cards or mahjong (OR=3.52, 95% CI: 1.91–6.47), current average time of physical activity ≤30 minutes per day (OR=8.66, 95% CI: 4.65–16.12), diabetic nephropathy (OR=1.95, 95% CI: 1.05–3.61) and stroke (OR=7.41, 95% CI: 3.41–16.11) were associated with a higher risk of CI in T2DM patients with hypertension. The area under the ROC curve of this model was 0.925 (95% CI: 0.902–0.949).

**Conclusions:**

The prevalence of CI was high in T2DM patients with hypertension in Hunan, China. Age, educational level, household income, current reading books or newspapers status, current playing cards or mahjong status, current average time of physical activity per day, diabetic nephropathy, and stroke were associated with CI in T2DM patients with hypertension.

## 1 Introduction

Type 2 diabetes mellitus (T2DM) and hypertension are commonly found to coexist due to similar risk factors such as endothelial dysfunction, vascular inflammation, and obesity (1). According to the latest Global Report on Hypertension released by the World Health Organization, the number of adults with hypertension doubled from 650 million in 1990 to 1.3 billion in 2019 (2). Furthermore, the total number of people with diabetes will rise to 643 million by 2030 and 783 million by 2045 (3). Previous meta-analyses showed that the pooled prevalence of hypertension among the diabetic population was approximately 50%, suggesting that there may be more than 322 million comorbidity cases worldwide in 2030 (4–6). Having one of these conditions increases the risk of developing the other by 1.5–2.0 times (7). Furthermore, such comorbidity confers a 2- to 4- fold increased risk of cardiovascular disease, end-stage kidney disease, and death (8–10).

Cognitive impairment (CI), characterized by the difficulty in processing thoughts, has become a major public health problem that reduces the quality of life of middle-aged and older adults (11–13). A growing body of evidence has suggested that the comorbidity of hypertension and diabetes can also accelerate cognitive decline (14, 15). A study in China reported that compared to those with hypertension only, individuals with both T2DM and hypertension had a higher risk of suffering from CI (odds ratio [OR]=1.44, 95% confidence interval [95% CI]: 1.14–1.80) (16), and Gorska-Ciebiada et al. (17) found that compared to those with T2DM only, individuals with both T2DM and hypertension had an increased risk of suffering from CI (OR=12.5, 95% CI: 2.92–54.0). The presence of CI in T2DM patients with hypertension may lead to poor self-management and medication adherence, ultimately contributing to increased mortality (18–20). Therefore, understanding the prevalence of CI in T2DM patients with hypertension and its associated factors is crucial, given that it cannot only facilitate the clinical management of such cases, but also help identify the high-risk group of CI among this population, as well as find intervention targets to reduce the incidence of CI.

Previous studies on this research topic have mainly focused on the general elderly populations, and those with either T2DM or hypertension (16, 21–24). These studies indicated that the prevalence of CI may differ by the sociodemographic, lifestyle and disease-related characteristics (16, 21–25). Specifically, sociodemographic characteristics included age, sex, and educational level (21, 24); lifestyle characteristics included smoking status, physical exercise, and reading status (16, 26); and disease-related characteristics included diabetes course, hypertension course, diabetic retinopathy, and cardiovascular disease (21, 24, 27). However, there is still a lack of research specifically addressing the prevalence of CI and its associated factors in T2DM patients with hypertension exclusively. Therefore, this study aimed to identify the prevalence of CI and its associated factors in T2DM patients with hypertension by comprehensively assessing the potential role of sociodemographic, lifestyle and disease-related characteristics.

## 2 Methods

### 2.1 Ethical approval

The study protocol was approved by the Ethics Committee of Xiangya School of Public Health, Central South University (No: XYGW–2021–27), and all participants provided informed consent.

### 2.2 Study design and populations

This cross-sectional study was performed at the Department of Endocrinology of the First People’s Hospital of Yuanjiang City, Hunan Province, China, from July 2022 to February 2023. The hospital is a secondary hospital located in Yiyang City, Hunan Province, China. A total of 475 consecutive T2DM patients with hypertension were included. The inclusion criteria were as follows: (1) diagnosed with T2DM according to the Guidelines for the Prevention and Treatment of Type 2 Diabetes Mellitus in China (2020 edition) (28); (2) diagnosed with hypertension according to the Guidelines for the Prevention and Control of High Blood Pressure in China (2018 Revision) (29); (3) aged ≧50 years; and (4) volunteered to participate in this study and signed an informed consent form. Patients with clinical dementia were excluded.

### 2.3 Data collection

Data on sociodemographic and lifestyle characteristics were collected via face-to-face survey by trained investigators who received uniformly standardized training prior to the interview. Diabetes- and hypertension- related information was extracted from medical records, and cognitive function were assessed by experienced physicians.

### 2.4 Outcome variable

The outcome of this study was CI, assessed using the Mini-Mental State Examination (MMSE) scale by experienced physicians. The MMSE was developed by Folstein et al. (30) and comprises 30 items and five cognitive domains including orientation, immediate memory, attention and calculation, recall ability, and language ability with a total score of 30 points. The cutoff values for detecting CI vary based on the educational level, and those with a score of ≤19, ≤22, and ≤26 for an educational level of illiterates, primary school, and junior high school or above were considered as CI cases (31).

### 2.5 Independent variables

The independent variables in this study included sociodemographic, lifestyle and disease-related characteristics.

#### 2.5.1 Sociodemographic and lifestyle characteristics

Sociodemographic characteristics included sex, age, marital status, educational level, per capita monthly household income, location of residence, residency status, work status, and body mass index (BMI). Lifestyle characteristics included smoking history, drinking history, current tea drinking status, current reading books or newspapers status, current computer users, current playing cards or mahjong status, and current average time of physical activity per day. Specifically, those who smoked continuously or cumulatively for more than 6 months or at least one cigarette per day were defined as having a smoking history; those who drank alcohol at least once a month for more than one year were defined as having a drinking history; those who drank tea ≥1 times/week in the past month were defined as current tea drinking; those who read ≥1 time/week in the past month were defined as current reading books or newspapers; those who used computers ≥1 time/week in the past month were defined as current computer users; and those who played cards or mahjong at least once in the past month were defined as current playing mahjong or cards.

#### 2.5.2 Disease-related characteristics

Diabetes-related characteristics included duration of diabetes, family history of diabetes, type of medication used for diabetes, and complications of diabetes (including diabetic nephropathy, diabetic retinopathy, diabetic foot, diabetic peripheral neuropathy, diabetic peripheral vascular disease, and diabetic ketoacidosis). Hypertension-related characteristics included duration of hypertension, family history of hypertension, and type of medication used for hypertension. Information on diabetes or hypertension comorbidities such as hyperlipidaemia, coronary heart disease, chronic kidney disease, fatty liver, and stroke was also extracted.

### 2.6 Statistical analysis

The data were analyzed with SPSS version 25.0 software. Mean ± standard deviation (SD) was used to describe normally distributed continuous variables, while median (inter-quartile range, IQR) was used to describe non-normally distributed continuous variables. Frequency (n) and proportion (%) were used to describe categorical variables. The *χ^2^* test or Fisher’s precision probability test was used to determine the univariable correlations between the independent variables and CI. Multicollinearity between the independent variables were assessed, and a variance inflation factor (VIF) value of less than 5 was considered as no multicollinearity problem. Backward stepwise multivariable logistic regression analysis was used to identify factors associated with CI by including the independent variables differed significantly in the univariable analyses. The goodness of fit of the model was evaluated using the Hosmer-Lemeshow goodness-of-fit test, with a *P* value of >0.05 indicated a good fit. In addition, the predictive value of the multivariable regression model was tested by the receiver operating characteristic (ROC) curve. All statistical tests were two-sided, and a *P* value of <0.05 was considered statistically significant.

## 3 Results

### 3.1 Participant characteristics

A total of 475 patients with complete data were included in this study, of which 198 (41.7%) were males and 277 (58.3%) were females. The age range of the study population was 50 to 95 years with a mean age of 69.80 ± 9.10 years. The mean duration of diabetes and hypertension was 13.36 ± 8.42 years, and 13.64 ± 8.63 years, respectively. The mean levels of fasting blood glucose (FBG), two-hour postprandial blood glucose (2hPG), and HemoglobinA1c (HbA1c, %) were 7.93 ± 2.42 mmol/L, 13.58 ± 3.30 mmol/L, and 8.12 ± 3.20, respectively. In addition, 82 (17.3%) attended high school and above; 199 (41.9%) had a BMI value of ≥24 kg/m^2^; 171 (36.0%) had a smoking history; 149 (31.4%) had a drinking history; 138 (29.1%) and 233 (49.1%) exhibited diabetic nephropathy and diabetic retinopathy, respectively; and 81 (17.1%) had a history of stroke. The characteristics of the study population are shown in **Tables 1**, **2**.

**Table 1:** Sociodemographic and lifestyle characteristics of the study population (n=475).

Variables	Category	Frequency (n)	Proportion (%)
Sex	Male	198	41.7
	Female	277	58.3
Age (year)	50–69	222	46.7
	≥70	253	53.3
Marital status	Unmarried	64	13.5
	Married	411	86.5
Educational level	High school or above	82	17.3
	Middle school	147	30.9
	Elementary school or below	246	51.8
Per capita monthly household income (RMB)	≤2,000	118	24.8
	>2,000	357	75.2
Location of residence	Urban area	294	61.9
	Rural area	181	38.1
Living alone	No	437	92.0
	Yes	38	8.0
Current work status	Employed	45	9.5
	Not employed	430	90.5
BMI	<18.5	36	7.6
	18–24	240	50.5
	24–28	156	32.8
	≥28	43	9.1
Smoking history	No	304	64.0
	Yes	171	36.0
Drinking history	No	326	68.6
	Yes	149	31.4
Current tea drinking status	No	394	82.9
	Yes	81	17.1
Current reading books or newspapers status	No	420	88.4
	Yes	55	11.6
Current computer user	No	455	95.8
	Yes	20	4.2
Current playing cards or mahjong status	No	273	57.5
	Yes	202	42.5
Current average time of physical activity per day (minutes)	≤30	256	53.9
	>30	219	46.1

**Table 2:** Disease-related characteristics of the study population (n=475).

Variables	Category	Frequency (n)	Proportion (%)
Duration of diabetes (year)	<5	83	17.5
	5–9	86	18.1
	10–14	105	22.1
	15–19	101	21.3
	≥20	100	21.1
Family history of diabetes	No	317	66.7
	Yes	158	33.3
Types of diabetes medication	No	25	5.3
	OAH	269	56.6
	insulin	90	18.9
	OAH+insulin	91	19.2
Diabetic nephropathy	No	337	70.9
	Yes	138	29.1
Diabetic retinopathy	No	242	50.9
	Yes	233	49.1
Diabetic foot	No	460	96.8
	Yes	15	3.2
Diabetic peripheral neuropathy	No	154	32.4
	Yes	321	67.6
Diabetic peripheral vascular disease	No	453	95.4
	Yes	22	4.6
Diabetic ketoacidosis	No	445	93.7
	Yes	30	6.3
Duration of hypertension (year)	<5	67	14.1
	5–9	93	19.6
	10–14	121	25.5
	15–19	85	17.9
	≥20	109	22.9
SBP (mmHg)	<120	60	12.6
	120–139	155	32.6
	≥140	260	54.8
DBP (mmHg)	<60	11	2.3
	60–89	341	71.8
	≥90	123	25.9
Family history of hypertension	No	315	66.3
	Yes	160	33.7
Types of hypertension medication	No	13	2.7
	Monotherapy	288	60.6
	Combination of drugs	174	36.6
Hyperlipidaemia	No	314	66.1
	Yes	161	33.9
Coronary heart disease	No	254	53.5
	Yes	221	46.5
Chronic kidney disease	No	427	89.9
	Yes	48	10.1
Fatty liver	No	376	79.2
	Yes	99	20.8
Carotid atherosclerosis	No	339	71.4
	Yes	136	28.6
Carotid plaque formation	No	367	77.3
	Yes	108	22.7
Stroke	No	394	82.9
	Yes	81	17.1

OAH, Oral Hypoglycemic Agent; SBP, Systolic blood pressure; DBP, Diastolic blood pressure.

### 3.2 Prevalence of CI in T2DM patients with hypertension

The mean score of MMSE was 25.89 ± 3.73, with scores ranging from 17 to 30. Based on the established cutoff values, 187 were considered as CI cases and 288 were considered as non-CI cases with normal cognitive function. The prevalence of CI in T2DM patients with hypertension was 38.9% (95% CI: 34.5%–43.3%).

### 3.3 Univariable analyses of factors associated with CI

The results of univariable analyses are shown in **Tables 3**, **4**. Sex, age, educational level, per capita monthly household income, location of residence, current work status, BMI, smoking history, current tea drinking status, current reading books or newspapers status, current computer users, current playing cards or mahjong status, and current average time of physical activity per day, duration of diabetes, diabetic nephropathy, duration of hypertension, coronary heart disease, fatty liver, and stroke differed significantly between the CI group and the non-CI group (*P*<0.05).

**Table 3:** Univariable associations of sociodemographic and lifestyle characteristics with CI.

Variables	Category	CI group (n=187, %)	Non-CI group (n=288, %)	*χ^2^*	*P* value
Sex	Male	64(34.2)	134(46.5)	7.060	0.008
	Female	123(65.8)	154(53.5)		
Age (year)	50–69	42(22.5)	180(62.5)	73.020	<0.001
	≥70	145(77.5)	108(37.5)		
Marital status	Unmarried	29(15.5)	35(12.2)	1.095	0.295
	Married	158(54.5)	253(87.8)		
Educational level	High school or above	6(3.2)	76(26.4)	103.985	<0.001
	Middle school	31(16.6)	116(40.3)		
	Elementary school or below	150(80.2)	96(33.3)		
Per capita monthly household income (RMB)	≤2,000	89(47.6)	29(10.1)	85.506	<0.001
	>2,000	98(52.4)	259(89.9)		
Location of residence	Urban area	82(43.9)	212(73.6)	42.579	<0.001
	Rural area	105(56.1)	76(26.4)		
Living alone	No	173(92.5)	264(91.7)	0.110	0.740
	Yes	14(7.5)	24(8.3)		
Current work status	Employed	4(2.1)	41(14.2)	19.347	<0.001
	Not employed	183(97.9)	247(85.8)		
BMI	<18.5	24(12.8)	12(4.2)	12.287	0.006
	18.5–24	88(47.1)	152(52.8)		
	24–28	58(31.0)	98(34.0)		
	≥28	17(9.1)	26(9.0)		
Smoking history	No	132(70.6)	172(59.7)	5.810	0.016
	Yes	55(29.4)	116(40.3)		
Drinking history	No	137(73.3)	189(65.6)	3.072	0.080
	Yes	50(26.7)	99(34.4)		
Current tea drinking status	No	169(90.4)	225(78.1)	12.027	0.001
	Yes	18(9.6)	63(21.9)		
Current reading books or newspapers status	No	182(97.3)	238(82.6)	23.889	<0.001
	Yes	5(2.7)	50(17.4)		
Current computer users	No	185(98.9)	270(93.8)	7.544	0.006
	Yes	2(1.1)	18(6.2)		
Current playing cards or mahjong status	No	156(83.4)	117(40.6)	84.967	<0.001
	Yes	31(16.6)	171(59.4)		
Current average time of physical activity per day (minutes)	≤30	161(86.1)	95(33.0)	128.706	<0.001
	>30	26(13.9)	193(67.0)		

**Table 4:** Univariable associations of disease-related characteristics with CI.

Variables	Category	CI group (n=187, %)	Non-CI group (n=288, %)	*χ^2^*	*P* value
Duration of diabetes (year)	<5	19(10.2)	64(22.2)	41.869	<0.001
	5–9	37(19.8)	49(17.0)		
	10–14	23(12.3)	82(28.5)		
	15–19	58(31.0)	43(14.9)		
	≥20	50(26.7)	50(17.4)		
Family history of diabetes	No	121(64.7)	196(68.1)	0.573	0.449
	Yes	66(35.3)	92(31.9)		
Types of diabetes medication	No	7(3.7)	18(6.3)	2.929	0.403
	OAH	103(55.1)	166(57.6)		
	insulin	41(21.9)	49(17.0)		
	OAH+insulin	36(19.3)	55(19.1)		
Diabetic nephropathy	No	120(64.2)	217(75.3)	6.871	0.009
	Yes	67(35.8)	71(24.7)		
Diabetic retinopathy	No	103(55.1)	139(48.3)	2.108	0.147
	Yes	84(44.9)	149(51.7)		
Diabetic foot	No	179(95.7)	281(97.6)	1.265	0.261
	Yes	8(4.3)	7(2.4)		
Diabetic peripheral neuropathy	No	63(33.7)	91(31.6)	0.227	0.634
	Yes	124(66.3)	197(68.4)		
Diabetic peripheral vascular disease	No	175(93.6)	278(96.5)	2.226	0.136
	Yes	12(6.4)	10(3.5)		
Diabetic ketoacidosis	No	179(95.7)	266(92.4)	2.164	0.141
	Yes	8(4.3)	22(7.6)		
Duration of hypertension (year)	<5	15(8.0)	52(18.1)	26.655	<0.001
	5–9	35(18.7)	58(20.1)		
	10–14	36(19.3)	85(29.5)		
	15–19	47(25.1)	38(13.2)		
	≥20	54(28.9)	55(19.1)		
SBP (mmHg)	<120	21(11.2)	39(13.5)	4.044	0.132
	120–139	53(28.3)	102(35.4)		
	≥140	113(60.5)	147(51.1)		
DBP (mmHg)	<60	4(2.1)	7(2.4)	2.007	0.367
	60–89	141(75.4)	200(69.5)		
	≥90	42(22.5)	81(28.1)		
Family history of hypertension	No	125(66.8)	190(66.0)	0.039	0.844
	Yes	62(33.2)	98(34.0)		
Types of hypertension medication	No	7	6	1.298	0.523
	Monotherapy	114	174		
	Combination of drugs	66	108		
Hyperlipidaemia	No	133	181	3.466	0.063
	Yes	54	107		
Coronary heart disease	No	82	172	11.481	0.001
	Yes	105	116		
Chronic kidney disease	No	163	264	2.528	0.112
	Yes	24	24		
Fatty liver	No	163	213	11.988	0.001
	Yes	24	75		
Carotid atherosclerosis	No	138	201	0.890	0.345
	Yes	49	87		
Carotid plaque formation	No	148	219	0.621	0.431
	Yes	39	69		
Stroke	No	129	265	42.514	<0.001
	Yes	58	23		

### 3.4 Multivariable analyses of factors associated with CI

**Figure 1:**
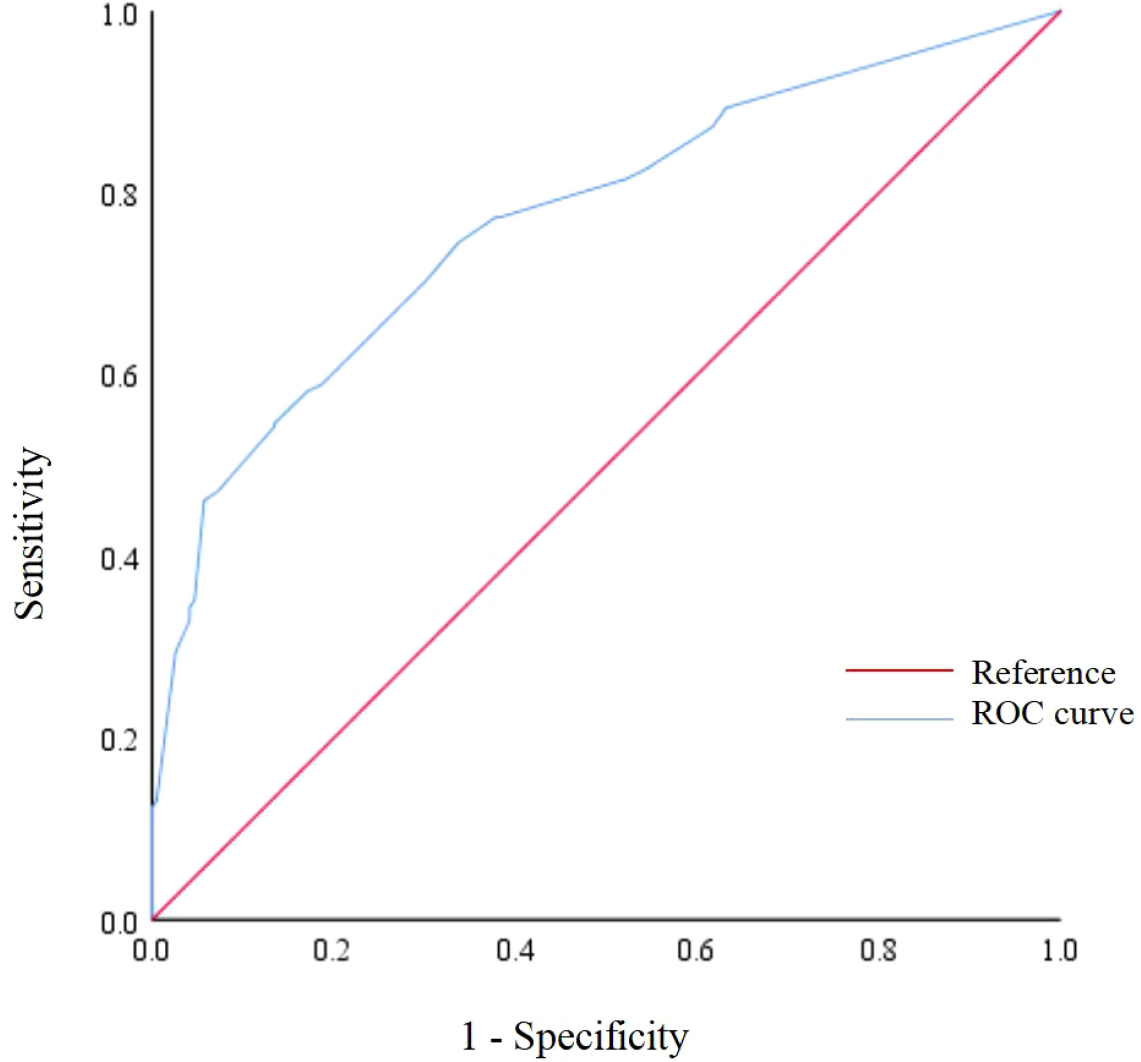
ROC curve of the multivariable logistic regression model.

The results of multivariable logistic regression analysis are presented in **Table 5**. Age, educational level, per capita monthly household income, current reading books or newspapers status, current playing cards or mahjong status, current average time of physical activity per day, diabetic nephropathy, and stroke were independently associated with CI in T2DM patients with hypertension. Aged ≥70 (OR=3.21, 95% CI: 1.74–5.93), elementary school or below (OR=3.51, 95% CI: 1.19–10.31), per capita monthly household income of ≤2000 RMB (OR=5.29, 95% CI: 2.66–10.51), not current reading books or newspapers (OR=4.48, 95% CI: 1.26–15.99), not current playing cards or mahjong (OR=3.52, 95% CI: 1.91–6.47), current average time of physical activity ≤30 minutes per day (OR=8.66, 95% CI: 4.65–16.12), diabetic nephropathy (OR=1.95, 95% CI: 1.05–3.61), and stroke (OR=7.41, 95% CI: 3.41–16.11) were associated with a higher risk of CI. The Hosmer-Lemeshow test indicated a good model fit (*P*=0.739). **Figure 1** shows the ROC curve of the multivariable model. The area under the ROC curve was 0.925 (95% CI: 0.902–0.949) with a *P* value of <0.001, indicating a good model fit.

**Table 5:** Multivariable logistic regression analysis between the independent variables and CI.

Variables	Category	b	SE	Wald-test	OR (95%CI)	*P* value
Age	50–69				1	
	≥70	1.17	0.31	13.85	3.21(1.74–5.93)	<0.001
Educational level	High school or above				1	
	Middle school	0.32	0.55	0.33	1.38(0.47–4.07)	0.565
	Elementary school or below	1.25	0.55	5.19	3.51(1.19–10.31)	0.023
Per capita monthly household income (RMB)	>2,000				1	
	≤2,000	1.67	0.35	22.56	5.29(2.66-10.51)	<0.001
Current reading books or newspapers status	Yes				1	
	No	1.50	0.65	5.35	4.48(1.26–15.99)	0.021
Current playing cards or mahjong status	Yes				1	
	No	1.26	0.31	16.38	3.52(1.91–6.47)	<0.001
Current average time of physical activity per day (minutes)	>30				1	
	≤30	2.16	0.32	46.41	8.66(4.65–16.12)	<0.001
Diabetic nephropathy	No				1	
	Yes	0.67	0.32	4.47	1.95(1.05–3.61)	0.035
Stroke	No				1	
	Yes	2.00	0.40	25.62	7.41(3.41–16.11)	<0.001

## 4 Discussion

This study investigated the prevalence of CI and its associated factors in T2DM patients with hypertension in Hunan Province, China. To our knowledge, this is the first study to identify the prevalence of CI and its associated factors in T2DM patients with hypertension exclusively. This study found that the prevalence of CI was 39.37% (95 CI%: 34.98%–34.98%), which was much higher than the estimates reported by previous studies focused on either T2DM patients or hypertension patients (32–34). This difference may be explained by the fact that the comorbidity of diabetes and hypertension can cause damage to the central nervous system, which can subsequently accelerate cognitive decline (35). Given the high prevalence of CI in T2DM patients with hypertension found in this study and the fact that CI is associated with poor medical compliance (36, 37), early identification of those at high-risk group of CI is needed.

Advanced age is a common risk factor for CI in the general population (38, 39). Consistently, this study found that advanced age increased the risk of CI in T2DM patients with hypertension, which may be attributed to aging-related decline in synaptic density and function in the brain (40–42). The current study also found that lower educational levels were associated with a higher risk of CI, which is consistent with the findings of several previous studies conducted in T2DM populations (43, 44). Education enhances brain reserve capacity to prevent brain damage by promoting synaptic density and maintaining adequate and stable cerebral blood flow (45). Furthermore, the study showed that low income was associated with a higher risk of CI (OR=5.29, 95% CI: 2.66–10.51), which is consistent with several studies conducted among older populations (46, 47). For example, a study conducted among older adults in Saudi Arabia found that low income was associated with cognitive decline (adjusted OR [aOR]=1.825, 95% CI: 1.17–2.86) (47). This may be explained by the fact that individuals with higher income may have greater social support, more medical resources, and higher health literacy (48). Therefore, those with advanced age, low educational level, and low income should be given special concern for the management of T2DM patients with hypertension in the clinical practice.

This study showed that current reading books or newspapers and current playing cards or mahjong were associated with a lower risk of CI in T2DM patients with hypertension, which is consistent with the finding of previous studies conducted among older populations (49, 50). For example, a cohort study of 10,318 older Australians found that reading books, newspapers, or magazines and playing games, cards, or chess were associated with a reduced risk of dementia over 10 years (49), and a study among Chinese older adults found that compared to those who “never” engaged in reading books or newspapers and playing cards or mahjong, those who engaged in such activities “almost every day” were at a decreased risk of CI, the fully-adjusted hazard ratios were 0.64 (95% CI: 0.53–0.78) and 0.70 (95% CI: 0.56–0.86), respectively (50). Intellectual activities such as reading books and playing games may lower the risk of dementia by improving cognitive reserve and increasing stress tolerance (49, 51). In addition, this study showed that the current average time of physical activity per day less than 30 minutes was associated with an increased risk of CI in T2DM patients with hypertension. The cognitive benefits of physical exercise are associated with increased plasticity and reduced inflammation within the hippocampus (52). A randomized controlled trial in the community showed that physical activity, particularly moderate-intensity aerobic exercise, enhanced cognitive function and health-related quality of life in older adults with CI (53). Therefore, the cognitive function of T2DM patients with hypertension could be maintained or improved by reading books or newspapers, playing cards or mahjong, and taking regular exercise.

This study showed that diabetic nephropathy, a microvascular complication of diabetes (54), was associated with a higher risk of CI. One possible mechanism is that brain and kidney damage are characterized by similar microvascular lesions, and that when renal function is impaired, it affects the cerebral microcirculation and the blood-brain barrier, which increases the risk of CI (55). This finding was also consistent with a previous study in T2DM patients (55). Our study adds significantly to the existing body of knowledge by showing that diabetic nephropathy could lead to decreased cognitive function in those with both T2DM and hypertension. Moreover, stroke is a major health problem worldwide and a leading cause of long-term disability and death (56). This study found that a history of stroke was associated with a higher risk of CI in T2DM patients with hypertension, which may be explained by the mechanisms related to inadequate cerebral perfusion, white matter damage, and neuroinflammation (57). Therefore, comprehensive management and treatment of T2DM complications and comorbidities are of great importance for maintaining the cognitive function of those with both T2DM and hypertension.

This study has several limitations. First, the cross-sectional study design did not allow for any causal relationships between the variables of interest. Secondly, this was a hospital-based study with a single center, which may limit the generalizability of the findings. Thirdly, recall bias remained an issue when collecting data. In addition, this study used only the MMSE to assess CI, which could result in misclassification compared to the gold standard. Therefore, future longitudinal studies with gold standard are needed.

## 5 Conclusions

The prevalence of CI in T2DM patients with hypertension was high in Hunan, China. Age, educational level, household income, current reading books or newspapers status, current playing cards or mahjong status, current average time of physical activity per day, diabetic nephropathy, and stroke were associated with CI in T2DM patients with hypertension. Therefore, regular reading, intellectual activity and physical activity should be considered as intervention targets for the prevention and management of CI, and those with advanced age, low educational level, and low household income should be given special concern. In addition, it is suggested to maintain or improve the cognitive function of those with both T2DM and hypertension via strengthening the management of diabetic complications and comorbidities including diabetic nephropathy and stroke.

## Data Availability

The raw data supporting the conclusions of this article will be made available by the authors, without undue reservation.
